# PAIN AMONG OLDER MEXICANS: FINDINGS FROM THE MEXICAN HEALTH AND AGING STUDY

**DOI:** 10.1093/geroni/igz038.2321

**Published:** 2019-11-08

**Authors:** Sadaf A Milani, Rafael Samper-Ternent, Martin Rodriguez, Rebeca Wong

**Affiliations:** University of Texas Medical Branch, Galveston, Texas, United States

## Abstract

In Mexico, palliative care and pain relief was recently added to the essential health services offered through Seguro Popular. Pain is more frequent in older adults, a growing segment of this population, and is a major contributor to decreased quality of life and increased morbidity. However, Mexico only has enough opioid analgesics to treat 36% of those in need. We used logistic regression models to examine correlates of pain using data from the 2012 wave of the Mexican Health and Aging Study, which includes Mexicans aged 50 and older (n=13,727). Overall, 38.2% of individuals reported that they often suffered from pain. Those who reported pain were more likely to be female (OR: 1.56; 95% CI: 1.41, 1.72), insured (OR: 1.17; 95% CI: 1.03, 1.33), live in a semi-rural locality (OR: 1.18; 95% CI: 1.04, 1.34), report their health as fair or poor (OR: 2.99; 95% CI: 2.73, 3.29), be a past smoker (OR: 1.17; 95% CI: 1.06, 1.29), have at least one ADL limitation (OR: 2.58; 95% CI: 2.27, 2.93), report depression (OR: 2.19; 95% CI: 2.02, 2.37), or report arthritis (OR: 2.74; 95% CI: 2.45, 3.07). Those who did not report pain were more likely to be widowed or have higher education. Diabetes, stroke, and cancer were not significantly associated with pain. Given that Mexico does not have the resources to treat over half of individuals living with pain, understanding the high burden of pain in this population is important to inform interventions and improve quality of life.

